# The effect of social media interventions on physical activity and dietary behaviours in young people and adults: a systematic review

**DOI:** 10.1186/s12966-021-01138-3

**Published:** 2021-06-05

**Authors:** Victoria A. Goodyear, Grace Wood, Bethany Skinner, Janice L. Thompson

**Affiliations:** School of Sport, Exercise and Rehabilitation Sciences, University of Birmingham, Birmingham, West Midlands B15 2TT UK

**Keywords:** Physical activity, Diet, Social media, Facebook, Instagram, Reddit, Twitter, Young adults, Systematic review, Narrative review

## Abstract

**Background:**

The objectives of this systematic review were to update the evidence base on social media interventions for physical activity and diet since 2014, analyse the characteristics of interventions that resulted in changes to physical activity and diet-related behaviours, and assess differences in outcomes across different population groups.

**Methods:**

A systematic search of the literature was conducted across 5 databases (Medline, Embase, EBSCO Education, Wiley and Scopus) using key words related to social media, physical activity, diet, and age. The inclusion criteria were: participants age 13+ years in the general population; an intervention that used commercial social media platform(s); outcomes related to changes to diet/eating or physical activity behaviours; and quantitative, qualitative and mixed methods studies. Quality appraisal tools that aligned with the study designs were used. A mixed methods approach was used to analyse and synthesise all evidence.

**Results:**

Eighteen studies were included: randomised control trials (*n* = 4), non-controlled trials (*n* = 3), mixed methods studies (*n* = 3), non-randomised controlled trials (*n* = 5) and cross-sectional studies (*n* = 3). The target population of most studies was young female adults (aged 18–35) attending college/university. The interventions reported on positive changes to physical activity and diet-related behaviours through increases in physical activity levels and modifications to food intake, body composition and/or body weight. The use of Facebook, Facebook groups and the accessibility of information and interaction were the main characteristics of social media interventions. Studies also reported on Instagram, Reddit, WeChat and Twitter and the use of photo sharing and editing, groups and sub-groups and gamification.

**Conclusions:**

Social media interventions can positively change physical activity and diet-related behaviours, via increases in physical activity levels, healthy modifications to food intake, and beneficial changes to body composition or body weight. New evidence is provided on the contemporary uses of social media (e.g. gamification, multi-model application, image sharing/editing, group chats) that can be used by policy makers, professionals, organisations and/or researchers to inform the design of future social media interventions. This study had some limitations that mainly relate to variation in study design, over-reliance of self-reported measures and sample characteristics, that prevented comparative analysis. Registration number: PROPSERO;CRD42020210806.

**Supplementary Information:**

The online version contains supplementary material available at 10.1186/s12966-021-01138-3.

## Background

Social media is positioned as a powerful medium to reach, influence and change physical activity and diet-related behaviours [1, 2]. For example, the recent World Health Organisation (WHO) Global Action Plan for Physical Activity identified the potential of social media to reach and target large audiences to promote physical activity engagement [3]. Similarly, Public Health England’s social marketing strategy emphasised the use of social media to target diverse groups more effectively, engage populations and support health-related behaviour changes [4]. Across international contexts there is also evidence that social media is being used in education, clinical, workplace and community settings to influence physical activity and dietary behaviours in young people and adults [2, 5, 6]. However, there is currently no guidance available for policy makers, professionals or organisations on how to responsibly and effectively use social media in physical activity and diet interventions [2, 7, 8], and there is little robust evidence on how social media interventions inform changes to behaviours related to physical activity and diet [9, 10]. As such, there is a gap in research and policy that leaves researchers, practitioners, and individual users ill-equipped to optimise the potential benefits of social media in promoting and supporting healthy physical activity and diet-related behaviours.

Interventions that improve physical activity and diet-related behaviours are of vital importance in optimising public health [11]. Engaging in regular physical activity and consuming a healthy diet can lead to a reduction in the burden of non-communicable diseases and improve daily functioning, mental health and wellbeing [11, 12]. However, one in four adults (age 18 and older) and three in four adolescents (age 11–17) worldwide do not currently meet the global recommendations for physical activity set by the WHO [3]. Globally, it is also estimated that poor diets are responsible for 22% of all adult deaths, with the main unhealthy dietary factors identified as high sodium intakes and low intakes of wholegrains and fruits [12].

The benefits of social media for physical activity and diet interventions are grounded in social media’s extensive reach and the affordances of interaction, information and entertainment [2, 6]. Over a third of the world’s population (38%) use social media sites such as Facebook, Instagram and WhatsApp [13], with high rates of social media use not confined to the young and/or specific ethnicities, cultures, genders and/or socio-economic groups [4, 14, 15]. Previous systematic reviews on social media use and/or health-related social media interventions identified that social media can positively impact physical activity and diet-related behaviours [7–10, 16, 17]. These reviews report numerous health-related benefits of social media use for behaviour change, including: increased interaction; more available, shared and tailored information; increased accessibility to health information; peer/social/emotional support; and health surveillance. There is therefore evidence to suggest that social media interventions may have the potential to inform changes to behaviours related to physical activity and diet.

While systematic reviews on social media interventions for physical activity and diet have been published [7–10, 16, 17], none have identified the characteristics of social media use that are associated with positive physical activity and diet-related outcomes. Furthermore, most of the published reviews focused on clinical settings and clinical population groups, and there is little evidence across non-clinical groups, ages, genders and other demographic factors [7–10, 16, 17]. In turn, our understanding of how best to design social media interventions to reach mass audiences, and how to tailor interventions to target the needs of specific demographic groups, is currently limited. Finally, previous systematic reviews on social media interventions have included research published prior to 2014 [7, 10, 17]. While these reviews provide important evidence on the potential of social media for behaviour change, these findings may quickly become irrelevant due to the exponential growth in social media use and access [14, 15], and the technological advancement of social media sites since 2014. Notably, there has been a systemic shift from text-based social media communication toward communication via images and videos, where opportunities for anonymity, multi-platform interaction and temporal content are dominant and contemporary uses of social media [2, 18, 19].

To address these limitations in the published literature, the objectives of this systematic review were to update the evidence base on social media interventions for physical activity and diet since 2014, analyse the characteristics of interventions that resulted in changes to physical activity and diet-related behaviours, and assess differences in outcomes across different population groups. The research question was: does the use of social media influence diet-related (eating or nutrition) and physical activity behaviours in young people and adults, and if so, how? The findings from this review can be used to inform the development of robust guidance on the design of social media interventions to increase their potential to elicit positive changes in physical activity and diet-related behaviours.

## Methods

### Protocol and registration

The protocol for this review was registered with the International Prospective Register of Systematic Reviews (PROPSERO; CRD42020210806). The report follows the PRISMA guidelines and checklist for systematic reviews [20].

### Eligibility criteria

For inclusion in this review, studies fulfilled the following PICOS statement:

- P (Participants): age 13 years and older in the ‘general population’ (i.e. non-clinical populations)
- I (Intervention): use of one or more commercial social media platforms: for example, Facebook, YouTube, Reddit, Twitter, Instagram, SnapChat, WhatsApp, Pinterest and TikTok
- C (Comparison): no engagement with or use of social media; or no comparison (such as in cross-sectional study designs)
- O (Outcomes): changes to diet/eating and/or physical activity knowledge, attitudes or behaviours
- S (Study Type): Quantitative, Qualitative or Mixed Methods studies, including randomised controlled trials (RCTs), non-randomised interventions, and observational studies.

Studies were included if they were peer-reviewed publications and were written in the English language. The exclusion criteria for this review included grey literature and articles reporting on clinical population groups. Clinical population groups refer to participants who were recruited from a medical facility and/or were receiving medical treatment. Studies reporting on participants who were obese or overweight and were not receiving medical treatment were included. Furthermore, articles were excluded if they reported on non-commercial social media platforms and bespoke social media platforms created specifically for the intervention. This review also applied a definition of social media [21] as platforms for interaction that require users to create a profile and involve the consumption and production of user-generated content; thus, papers reporting on video conferencing platforms such as Zoom or Skype were excluded.

### Search strategy

Five electronic databases were searched, including Medline, Embase, EBSCO Education, Wiley and Scopus, between December 2020 and February 2021. Titles, abstracts and keywords were searched using the following terms: 1) Social media (e.g. “social media”, “social network”, Facebook, Instagram, etc); 2) Physical activity (e.g. “physical activity”, Exercise, workout, etc); 3) Diet (e.g. Diet, Nutrition, “Dietary Behaviour”, Eating, etc); 4) Age Groups (e.g. “High School” “Young Adult”, “Adult’, etc). A full overview of the search terms is provided in the Supplementary materials. Articles retrieved from the databases were limited to those published from 2014 onwards. This limitation was in place due to: a) previous reviews including peer review articles up until this time point [7, 10]; b) the exponential change in social media from 2014 onwards; and c) the types of social media adopted and used in society expanding from 2014 onwards, shifting from predominantly Facebook toward more interactive and visual forms of social media, including Instagram, WhatsApp and YouTube [14, 18].

### Data extraction

**Fig. 1:**
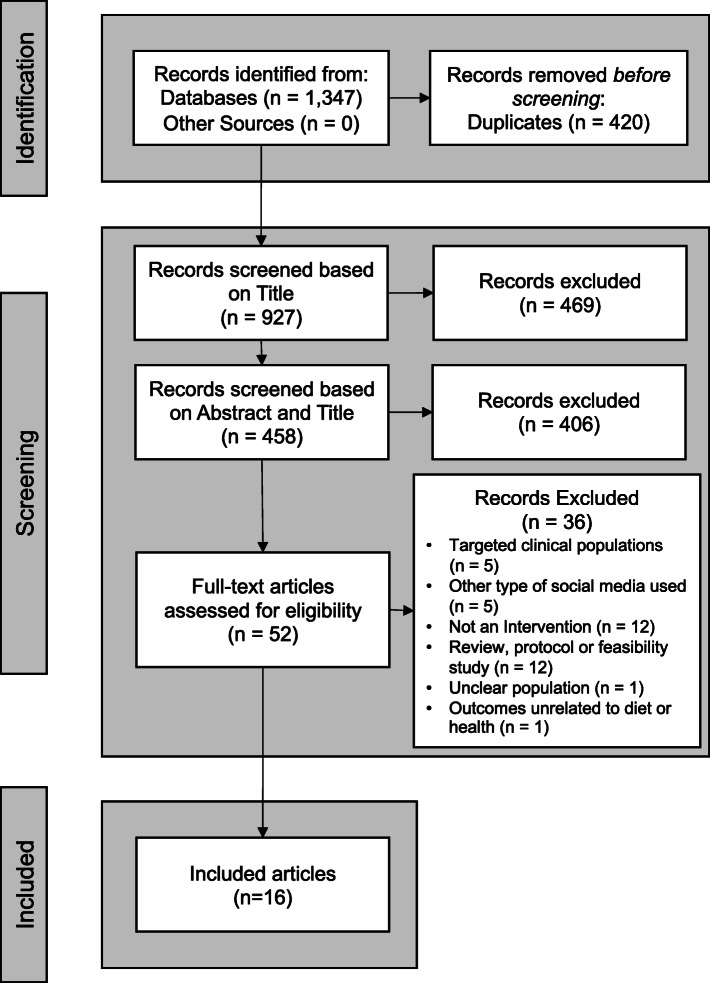
PRISMA Flow Diagram of Search Strategy

All papers from the database search were collated using EndNote (X9, 2019) reference management software. The screening processes were completed independently by three reviewers (authors GW, BS and VG), with conflicts or undecided articles reviewed by a fourth reviewer (author JLT) for their inclusion or exclusion. Titles were initially screened, followed abstract screening and articles were excluded if they did not meet the inclusion criteria. Full text reviews of articles were then completed by three reviewers independently (GW, BS and VG) with the reasons for exclusion recorded (Fig. 1). Article data were extracted from the final set of included articles by one reviewer (GW), and checked independently by two reviewers (VG and BS). The data extracted included: 1) Details of Intervention (e.g. aims, study design, setting); 2) Participant characteristics; 3) Methods (e.g. general overview of methods, social media used and defined, context and forms of social media use, type of data, etc); 4) Outcomes (e.g. Health outcomes for physical activity or diet, social media outcomes, forms of social media use, and associations between social media and health outcomes); and 5) Key Conclusions, including limitations and key recommendations.

### Assessment of quality

Included articles were critically assessed for quality and bias. The Integrated Quality Criteria for Review of Multiple Study Designs (ICROMS) tool [22] was utilised for the multiple study designs for included articles. Mixed-methods studies were assessed using the Mixed Methods Appraisal Tool [23], and the three cross-sectional studies were assessed using the Joanna Briggs Institute Critical Appraisal checklist for analytical cross-sectional studies [24]. Articles were grouped and weighted based on the outcomes from these three tools. All studies were independently rated by GW, BS and VG, and disagreements resolved through discussion with JLT.

### Data analysis

The study designs, outcome measures and variables for physical activity and diet varied across the studies. As such, it was not possible to perform a meta-analysis. A narrative synthesis was completed following Cochrane guidelines [25]. Findings from quantitative and mixed methods studies were described numerically and/or textually to provide a summary of evidence on the: (i) characteristics of the social media interventions; and (ii) effects on physical activity and diet. Relationships were then qualitatively examined between studies with the aim of identifying factors that related to intervention effectiveness, such as characteristics of social media use, intervention design, and variability in populations.

## Results

### Description of studies and quality assessment

Sixteen papers were reviewed and these included 18 studies, and are summarised in Table 1. With regards to design, 4 studies were randomised control trials (RCTs) [26–29], 3 mixed methods [30–32], 3 non-controlled before-after trials (NCBA) [33–35], 5 controlled before-and-after trial (CBA) [36–40], and 3 cross-sectional [37, 39, 41]. Most of the papers were published between 2017 and 2020 (13 of 16) [26–35, 37, 40, 41], and included the following social media platforms: Facebook, Instagram, Reddit, Twitter, and WeChat. Most data were collected within the USA (9 studies) [28–30, 33–37, 39], and 11 out of 18 studies targeted young adults aged between 18 and 35 years old [26, 28, 29, 33, 36–40] and eight included college/university students [28, 33, 36–40]. Five of the 18 studies targeted specific populations including primigravid women [34], adults at risk of colorectal cancer [30], nurses [32], and overweight or obese individuals [27, 35].

**Table 1:** Summary of Included Studies

Author	Location of Research	Sample	Design	Physical Activity and/or Diet Measures	Characteristics of Social Media Intervention and Control Groups	Main Findings
Ashton et al., (2017) [27]	Australia	*N* = 50 (100% Male) Mean Age (SD): Intervention Group = 22.4 (2.0), Waitlist Control Group = 21.9 (2.1) Ethnicity = Not reported Target group = Young adult men aged 18–25 years	RCT	Physical Activity Level (Pedometer), MVPA (Self-reported), Diet Quality & Energy Intake (Australian Eating Survey FFW), Weight, Fat Mass, Skeletal Muscle Mass (Bioelectrical impedance analysis), BMI (stadiometer and weight), Waist Circumference (Tape Measure), systolic and diastolic blood pressure (composite measures), rest heart rate (automatic sphygmomanometer)	*Medium*: Facebook *Type of Social Media*: Information & Interaction - Facebook groups to facilitate social support, notifications for new materials and reminders for sessions. *Control Group*: continued normal routine and started program after a 3 month delay. *Duration:* 3 months	Statistically significant differences for the social media intervention: MVPA (*p* < 0.05, d = 0.58), daily vegetable servings (*p* < 0.05, d = 0.62), percentage energy from ED- NP* foods (*p* < 0.01, d = 0.73), weight loss (*p* < 0.05, d = 0.63), percentage weight loss (*p* < 0.05, d = 0.67), waist circumference (*p* < 0.001, d = 0.89), BMI* (*p* < 0.01, d = 0.81), body fat mass (*p* < 0.05, d = 0.67), plasma total cholesterol (*p* < 0.05, d = 0.60), LDL* cholesterol (*p* < 0.01, d = 0.83) and ratio of total cholesterol-to-HDL* cholesterol (*p* < 0.05, d = 0.60)
Jane et al. (2017) [28]	Australia	*N* = 67 (85% Female; 15% Male) Mean Age (SD): Control Group = 50.2 (2.4); Pamphlet Group = 54.1 (2.3); Facebook Group = 47.0 (2.3) Ethnicity = Not reported Target group = Individuals with a BMI between 25 and 40 kg/m^2^ (overweight or obese)	RCT	Weight (digital scales), height (stadiometer), lean mass and fat mass (bioelectrical impedance), waist and hip circumference, blood pressure (sphygmomanometer), fasting blood glucose (glucometer), blood lipids insulin (blood test) all measured at clinical appointments at 6, 12, 18 and 24 weeks. Dietary intake, physical activity and step count (from self-reported three-day food, physical activity and step count records)	*Medium:* Facebook *Type of Social Media:* Information and Interaction - Instructions for specified diet (Total Wellbeing Diet) shared, access to weight management program and interaction with other members via Facebook. *Control Group*: received standard care *Comparison Group:* received intervention through pamphlets *Duration:* 24 weeks	Intervention group had statistically significant greater weight loss than the control group (group 1) at 6 weeks (− 2.7%, *p* = 0.01 and − 2.5%, *p* = 0.02 respectively), 18 weeks (− 4.5%, p = 0.02 and − 4.9%, p = 0.02 respectively), and 24 weeks (− 3.6%, *p* = 0.05 and − 4.8%, p = 0.01 respectively). Social media intervention had significant reduction in BMI and waist circumference at 18 weeks (− 1.6 kg/m^2^, *p* = 0.04 and − 1.5 kg/m^2^, *p* = 0.04 respectively) and 24 weeks (− 1.5 kg/m^2^, *p* = 0.02). Statistically significant increase in energy expenditure at 6 weeks (+ 588.8 kJ/day, *p* = 0.03). There was no statistically significant difference between the groups for step count and fat, alcohol or mean energy intake.
Pope et al. (2019) [29]	USA	*N* = 38 (74% Female; 26% Male) Mean Age (SD): Intervention Group = 21.2 (4.0); Control Group = 21.8 (2.8) Ethnicity: Non-Hispanic White *N* = 27 Asian *N* = 11 Target Group = College students aged 18–35 years, BMI > 18.5 kg/m^2^	RCT	Physical Activity (accelerometers for 7 days at baseline, 6 weeks and 12 weeks), Cardiorespiratory fitness (YMCA 3-min step test at baseline and 12 weeks), Height, weight and body composition (stadiometer at baseline and 12 weeks and bioelectrical impedance scale), Dietary behaviours (ASA24 food recall three times at baseline, 6 and 12 weeks).	*Medium*: Facebook *Type of Social Media:* Information - education and health tips were provided twice a week in a Facebook group and participants used a smartwatch to track their physical activity. *Control Group*: No smartwatch but access to the Facebook Group. *Duration:*12 weeks	MVPA from baseline to 6 weeks increased for both intervention (4.2 min) and control (1.6 min) groups (not statistically significant). Decreased daily caloric intake for both groups (intervention = − 41 cal, control = − 143.3 cal) not statistically significant) and decreased vegetable consumption at 6 and 12 weeks for intervention group (− 0.2 cups each time) (not statistically significant).
Vogel et al. (2019) [30]	USA	*N* = 500 (54.6% Female; 44.8% Male; 0.6% Other) Mean Age (SD) = 20.9 (2.0) Ethnicity: Non-Hispanic Caucasian *N* = 366 Native American *N* = 5 African American *N* = 13 Asian/Pacific Islander *N* = 6 Hispanic *N* = 34 Multiple Ethnicities *N* = 72 Target Group = Young Adults Aged 18–25 years, current smoker and current Facebook user	RCT	Self-reported questionnaire on diet: (healthy eating, fruit and vegetable consumption) physical activity (regular exercise engagement and MVPA) and latent class analysis on health risk behaviours (high-fat diet, low fruit and vegetable consumption, physical inactivity, poor sleep hygiene, poor stress management, heavy drinking and substance use)	*Medium*: Facebook *Type of Social Media:* Interaction and Information – information about smoking cessation was posted to a private Facebook group once per day and a live counselling session was held once a week on Facebook. *Control Group:* Referred to smokefree.gov. *Duration:* 90 days with data collected at baseline, and at 3, 6 and 12 months.	The Facebook intervention resulted in smoking abstinence. A significant direct effect of 3 month smoking abstinence on lower likelihood of 12 month metabolic risk (*P* < 0.010). This effect was mediated by readiness to increase fruit and vegetable consumption at 6 months (*P* < 0.05). A significant and direct effect of 3 month smoking abstinence and readiness to increase fruit and vegetable consumption at 6 months (*P* = < 0.03). Overall, readiness to increase fruit and vegetable consumption mediated the relationship between abstinence from smoking and decrease metabolic risk behaviours.
Chung et al., (2017) [34]	USA	*N* = 12 (67% Female; 33% Male) Mean Age: Overweight/Obese Group = 20.3; Healthy weight Group = 19.0 Ethnicity: Caucasian N = 6 African *N* = 4 Asian American *N* = 1 American Indian *N* = 1 Target group = College students (age ≥ 18 years), BMI categorised as overweight/obese (BMI 25–34.9 kg/ m^2^) or healthy weight (BMI 22.5–24.9 kg/ m^2^)	NCBA (Single arm intervention pilot study)	Weight and body fat percentage (Fitbit aria wireless scale), intake of fruit, vegetables and SSB* (self-reported), any lifestyle changes (self-reported)	*Medium*: Twitter *Type of Social Media:* Information, Interaction and Gamification – Private Twitter group and use of a FitBit; tweets between participants and researchers on diet and physical activity, personalised feedback and group challenges with prizes. *Intervention Design*: Participants were divided into 2 groups: overweight/obese and health weight *Duration:* 3 months	Participants had a decline in steps on weekends and during holidays but increased with 1 day challenges and were sedentary for the majority of their day (not statistically significant). An increase in fruit intake was seen for 92% of participants and vegetable intake for 58% (self-reported, not statistically significant). Overweight participants lost 1–5 pounds and healthy weight participants lost 0.2 to 7 pounds (not statistically significant).
Williams et al. (2019) [35]	USA	*N* = 23 (100% Female) Mean Age = Not reported Ethnicity = Not reported Target Group = Primigravid women, ≥18 years of age, English or Spanish speaking, receiving care at clinic	NCBA (Feasibility Study Pilot intervention)	Anthropometric measures (height, weight, weight gain during pregnancy, postnatal weight loss, baby length/weight), nutrition and physical activity (pre-post surveys).	*Medium*: Facebook *Type of Social Media:* Interaction and Information – Health messages were shared and interaction was promoted between the participants as well as with the health care worker. *Control Group*: No control group *Duration:* 3 years	Intervention was well-received and would be recommended to a friend. Postpartum effects: 70% (5 out of 7) of participants dropped below or within 5 pounds of their pre-pregnancy weight at 5 months postpartum (not statistically significant). Barriers to engaging in physical activity (PA) included no previous PA prior to pregnancy, beliefs or concerns about PA during pregnancy, lower motivation to attend PA class than compared to maternal nutrition/baby information, PA sessions were in a different location, and not all wanted to engage in group walking.
Willis et al. (2017) [36]	USA	*N* = 70 (84% Female; 16% Male) Mean Age (SD) Intervention Group = 46.8 (13.2) Mean Age (SD) Control Group = 47.6 (11.7) Ethnicity = Not specifically defined; 24% of total sample categorised as “Minorities” Target Group = Adults with obesity	NCBA (Randomized Feasibility Study)	Self-monitoring once per week: body weight (self-reported through scales), diet consumed and minutes of PA (using MyFitnessPal app), steps (using Fitbit activity monitor). Outcome Measures were collected in the laboratory by trained staff at baseline and month 6: body weight (digital scales), height (stadiometer) and waist circumference (tape measure). Diet Intake: log all food and beverage in the MyFitnessPal app for 3 consecutive days prior to laboratory visit which was downloaded by research staff. Physical activity: sub-set of participants wore an accelerometer for 7 days.	*Medium*: Facebook *Type of Social Media:* Interaction and Information- Health education lessons and audio recordings were posted and participants commented or posted four times a week (mandatory) on a message board. *Control Group:* Received same intervention by phone calls rather than through an online social network. *Duration:* 6 months	No statistically significant different for intervention and control group for weight change (kg), BMI, waist circumference, weight gain, weight loss, minutes of physical activity or steps.
Key et al. (2020) [31]	USA	*N* = 56 (66% Female; 35% Male) Mean Age (SD) = 58 (6) Ethnicity: 100% White Target group = Age ≥ 50 years, at risk for CRC*, one or more modifiable CRC risk factors	Mixed-Methods	Physical Activity (Fitbit device and self-reported), BMI (collected pre/post intervention), CRC screening status (questionnaire), Dietary Patterns (Vio-FFQ*), Diet Quality (Healthy Eating Index) Inflammatory effect on diet (Dietary Inflammatory Index)	*Medium*: Facebook *Type of Social Media:* Information and Gamification - Daily posts related to nutrition, physical activity and CRC factors and weekly challenges (to increases steps, get most likes) with prizes. *Control Group:* N/A *Duration:* 12 Weeks	The Healthy Eating Index Scores had a significant increase from baseline to post-intervention (*p* < .001), with the mean score increasing by 9 points (49.9 ± 9.9 to 58.6 ± 12.1). Dietary Inflammatory Index scores had a significant decrease from baseline to post-intervention (*p* = .002) from 2.8 ± 1.1 to 1.6 ± 1.7 BMI had no significant changes. The steps per day decreased − 33.0 between pre- (2007.1 to 1974,5 and post-intervention (not statistically significant).
Pappa et al. (2017) [32]	Not Stated	*N* (Total sample) = 107,886 *N* (Subset of platform users with gender, age, and weight change data available) Female) = 754 (56% Female; 44% Male) Mean Age (SD) = 25 (6) Ethnicity = Not reported Target Group = Users of sub-Reddit community called ‘LoseIt’	Mixed-Methods (Randomised Cross-sectional Survey)	Weight, weight check-ins (start, current and goal weight),age gender, height (self-reported) and BMI (calculated)	*Medium:* Reddit (Subreddit ‘LoseIt’ community) *Type of Social Media:* Information and Gamification - User activity on the subreddit group based on content users posted and interactions with other users. *Control Group:* N/A *Duration:* 4 years	3.7% (28/754) of users gained weight (mean 3.88%, SD 4.04), 3.5% (25/754) maintained weight, and 92.9% (701/754) lost weight. 514 of 754 users (68.2%) moved to a healthier weight category (i.e. high obese, moderate obese, low obese, overweight, normal) while part of the LoseIt community (not statistically significant).
Torquati et al. (2018) [33]	Australia	*N* = 47 (87% Females; 13% Male) Mean Age (SD) = 41.4 (12.1) Ethnicity = Not reported Target Group = Registered Nurses aged > 18	Mixed-Method (Pilot Intervention)	Changes to physical activity behaviour including MVPA, light activity, sedentary and steps per day (accelerometers), diet behaviour and dietary patterns (FFQ* Australian Eating Survey; Australian Recommended Food Score), chronic disease risk markers (weight, BMI, waist circumference and blood pressure), health, diet and physical activity self-efficacy (self-reported)	*Medium:* Facebook *Type of Social Media:* Information and Interaction – information and education content was shared through posts. *Control Group:* N/A *Duration:* 12 weeks	MVPA and daily steps decreased at 3 months (*P* = .01 and .04, respectively) and MVPA further decreased at 6 months. A significant time × interaction effect for MVPA and average daily steps (*P* = .01 and .05, respectively). Intake of fruit and vegetables improved significantly at 3 months and decreased slightly at 6 months (*P* = 0.04)
Wang et al. (2021) [39]	China	*N* = 110 (59% Female; 41% Male) Median Age (SD) = 18(1.0) (Intervention Group), 18(1.0) (Control Group) Ethnicity: Not reported Target Population: College Students	CBA (Non-randomized Control Intervention)	Diet (dietary intake estimates at baseline, logging meals and sharing food pictures), food intake (Self-reported), physical activity (physical activity at baseline, recommended to exercise 150 min/week and share this on the WeChat community), completion of daily tasks (self-check questionnaire), physical fitness (i.e. push-ups, squares, planks), body composition, waist, resting metabolic rate (completed at baseline and 21 days after)	*Medium:* WeChat and Zhishi mini-program *Type of Social Media:* Information, Interaction and Gamification– (i) daily health-related information shared, (ii) discussions initiated on health education content, sharing food pictures and physical activity performance in the WeChat group, (iii) mini exercise challenges were assigned. *Control Group:* Same as intervention but did not receive health-related information *Duration*: 21 days	Daily food intake for the intervention group significantly improved after 21 days in comparison to baseline (*P* < 0.05) for vegetable, fruit, milk and dairy products. Frequency intake of soybean (and related soybean products) significantly increased in the intervention group (*P* = 0.030). A significant enhancement of physical activity was shown for the intervention group, with 48 having low physical activity at baseline and 26 having a higher level at the end of the intervention (*P* = 0.004). Better changes in physical fitness were shown for the intervention group, with the length of squat time (*P* = 0.005) and plank time (*P* = 0.008) of the intervention group better than the control group. No food intake changes were shown for the control group between baseline and 21 days (*P* > 0.005)
Krishnamohan et al. (2017) [41]	India	*N* = 45 (47% Female; 53% Male) Age Ranges: Control Group 18–23); Intervention Group 18–23 N (Ethnicity) = Not reported Target group = First or Second Year Clinical University Students with a BMI ≥23 kg/m^2^	CBA	Changes in physical activity and diet patterns such as intake of fruits, vegetables and junk food (WHO STEPS questionnaire), BMI (scales and stadiometer), changes in weight (measurement not reported).	*Medium*: Facebook *Type of Social Media:* Information - Posting health education messages and content to participants three times a week. *Control Group*: 2nd year students with no access to social media *Duration:* 6 weeks	Statistically significant decrease in mean number of days per week of junk food intake reduced for both control (2.91 to 2.65 days) and intervention (3.27 days to 2 days) (*P* < 0.01). Significant decrease in BMI for those in the control group (25.57 to 25.15) (*P* < 0.05). BMI increased for those in the intervention group (26.66 to 26.74) (not statistically significant). Increase in mean minutes per week of moderate physical activity (9.32 min to 12.27 min) and for physical activity during travel (298.18 min to 496.36 min) for intervention group (not statistically significant). Increase in mean minutes per week for physical activity during travel at follow up for control group (761.52 min to 1036.52 min) and intervention group (298.18 min to 496.35 min) (not statistically significant).
West et al. (2016) [37]	USA	*N* = 58 (81% Female); 19% Male) Mean Age (SD) = 21.6 (2.2**)** Ethnicity = 90% white; other ethnicities not reported Target Group = Students who were registered for undergraduate courses in health	CBA (Controlled Quasi-experimental Study)	Weight (self-reported through Wi-Fi scales provided), Physical activity (physical activity tracker for steps and miles walked), BMI (digital scales), Behavioural Weight Control Practices (28 item checklist)	*Medium:* Facebook *Type of Social Media:* Interaction and Information- content related to how to maintain a healthy weight content was shared and posted, and group members interacted with one another. *Control Group:* Used Facebook but did not receive information on healthy weight maintenance. *Duration*: 9 Weeks	A significant increase in weight control strategies postintervention was observed for intervention group (− 1.1 ± 3.4; *P* = .003). These included self-weighing (*P* = .005), cutting out snacking (*P* = .001), reducing carbohydrate intake (*P* = .02), graphing weight (*P* = .01), reducing calorie intake (P = .02), reducing fat intake (P = .02), and increasing exercise (P = .02) Overweight students in the intervention group lost 1.8 ± 0.7 kg over 9 weeks in comparison to overweight students in the control group who lost 1.4 ± 1.7 kg (not statistically significant).
Mabe et al. (2014) [40]	USA	*Study 1* *N* = 960 (100% Female) Mean Age (SD) (Autumn) = 18.44 (0.85) Mean Age (SD) (Spring) = 19.10 (1.11) Ethnicity = 18.5% Hispanic; 86.5% White *Note: these are the percentages reported in the paper; N of participants in each ethnic group not reported. *Study 2* N = (Study 2) = 84 (100% Female) Mean Age (SD) = 18.39 (SD) Ethnicity: White = 77.4%; 15.5% Hispanic; 7.1% African American Target group = Female College Students who used Facebook weekly	Cross-Sectional (Study 1) and CBA (Study 2)	*Study 1* Eating attitudes and behaviours (EAT-26), duration of Facebook use (amount of time spent on Facebook per week). *Study 2* Eating attitudes (EAT-26).	*Study 1* *Medium:* Facebook *Type of Social Media:* cross sectional study exploring duration of Facebook use Study 2 *Medium:* Facebook *Type of Social Media*: General -participants were asked to spend 20 min on Facebook Control Group: 20 min on Wikipedia researching the Ocelot and related YouTube videos. *Duration:* 20 min	Study 1 – Significant positive correlation between duration of Facebook use and disordered eating for participants in fall (r(623) 5 .11, *p* < .01) and spring (r(334) 5.16, *p* < .01). Study 2 –EAT-26 scores were significantly associated with several Facebook items; participants with greater disordered eating were shown to endorse importance of receiving comments on status (r(83) 5 .32, *p* < .01), photos (r(83) 5 .29, p 5 .01), and receiving likes (r(83) 5 .29, *p* < .01), comparing their photos with female friends’ photos more often (r(83) 5 .22, p 5 .04). The urge to exercise significantly decreased for those spending 20 min on the internet (*p* < .001, d 5 .26), with the effect not dependent on the condition (*p* = .46), suggesting that this is for general internet use only and not Facebook use.
Raggat et al. (2018) [42]	Australia	*N* = 180 (84% Female; 15% Male; 1% Other) Median Age = 23.0 (IQR 19.0, 28.5) Ethnicity = Not reported Target Group = Aged > 16, residing in Australia, engaged with fitspiration content	Cross-Sectional	Disordered Eating Behaviours (Eating Attitudes Test-26), compulsive exercise behaviours (Exercise Addiction Inventory), perceived influence of fitspiration on health and well-being (online survey).	*Medium:* Any Social Media Platform *Type of Social Media:* Information and Interaction – participants were surveyed on the fitspiration* content they accessed, posted about or discussed with other users. *Control Group*: N/A *Duration*: 6 weeks	Fitspiration content inspired participants (*n* = 159, 90.3%) to exercise or eat healthy. Fitspiration content influenced health behaviours and beliefs through setting the ‘healthy ideal’, failure to achieve the ‘ideal’, being part of a community, and access to reliable health information.
Wicks and Keel (2020) [38]	USA	*Study 1* *N* = 2485 (76% Female; 24% Male) Mean Age (SD) = 19.01(1.80) Ethnicity = White (77%), Black (10%), Asian (4%), Hispanic (23%), Other (2%) Target Group = Male and Female College Students *Study 2* *N* = 89 (93% Female, 7.5% Male) Mean Age (SD) = 18.71(.97) Ethnicity = White (91%), Black (8%), Asian (6%), Hispanic (24%), Other (1%) Target Group = Individuals who endorsed posting edited photos in Study 1.	Cross-Sectional (Study 1) and CBA (Study 2)	*Study 1* Questionnaire to assess if Instagram is used and if photo editing applications are used to edit photos, Eating Attitudes Test-26 for disordered eating. *Study 2* Participants viewed their own photo (taken by researchers) for 1 min then completed Visual Analogue Scales before and after being assigned to one of four group conditions. Participants also completed a post- questionnaire battery (EAT-26, Body Shape Questionnaire, Trait Subscale of the State-Trait Anxiety Inventory, Centre for Epidemiological Studies Depression Scale and Instagram Survey). After 24 h, participants completed VAS and Follow-up Social Media Questionnaire.	Study 1 *Medium:* Instagram *Type of Social Media: Interaction* through posting their own photo to Instagram Study 2 *Medium:* Instagram *Type of Social Media: Interaction* *Group Conditions:* (1) Edited and posted photos as normal); (2) Edited photo but did not post; (3) Completed neutral questionnaire and posted unedited photos; (4) Completed neutral questionnaire and did not post photo. *Duration –* 24 Hours	Study 1 Participants who posted edited photos significantly differed on gender (X2[2] = 146.93, *p* < .001),where women endorsed posting editing photos compared to men, and race (X2[5] = 26.22, *p* < .001) where white and Asian individuals endorsed posting and editing photos compared to black individuals. Those who posted edited photos had a significantly higher likelihood of scoring above EAT-26 cut off for probable eating disorders compared to those who don’t (10.0 vs. 5.5% X2[1] = 12.63, *p* < .001). *Study 2* There were significant increases in eating disorder cognitions for those who posted photos (t[40] = 2.00, *p* = .05; d = .64).The posted edited photos conditions caused significant increases in eating disorder cognitions (t [18] = 2.36, *p* = .03); d = 1.11); the edit only condition caused significant decreases in eating disorder cognitions (t [20] = − 1.24, p = .03; d = .56). For disordered eating urges, significant post x time interaction where urges decrease significantly in those who did not post photos (t[40] = 1.22, *p* = .04; d = .39). From baseline to 24 h follow-up, there were significant decreases in eating disorder cognitions for participants (F[1, 76] = 7.36, *p* =. 008; partial n2 = .09) and significant decreases in anxiety (F[1,76] = 32.68, *p* < .001; partial n2 = .30) regardless of condition.

*SD Standard deviation, *IQR* inter-quartile range, *ED-NP* Energy Dense, Nutrient Poor, *BMI* Body Mass Index, *LDL* Low Density Lipoprotein, *HDL* High Density Lipoprotein, *SSB* Sugar-Sweetened Beverage, *CRC* Colorectal Cancer, Fitspiration is a popular social media trend containing images, quotes and advice for healthy eating and exercise, *FFQ* Food Frequency Questionnaire

The outcomes of the quality assessment are reported in Table 2. Twelve out of the 18 studies scored high and/or met all the quality assessment criteria for the relevant assessment tool [26–32, 36–41]. Of these papers, all articles reporting on RCT, CBA and mixed methods studies were included. Six of the 18 studies scored low and/or did not meet all of the quality assessment criteria for the relevant assessment tool [33–35, 37, 39, 41] and these included all cross sectional and NCBA studies. These studies were deemed as lower quality due to insufficient information being provided on one or more of the following: aims or objectives, justification for research design, sampling, bias, data collection measures, and ethics. For full details see Supplementary File A.

**Table 2:** Quality Assessment Score of Included Studies

Author, Year	Quality Assessment Tool	Study Design Category in Tool	Score/ Outcome Reviewer 1 (author – BS or VG)	Score/Outcome Reviewer 2 (author - GW)
Ashton, 2017 [27]	ICROMS	RCT	26	27
Chung, 2017 [34]	ICROMS	NCBA	19	21
Jane, 2017 [28]	ICROMS	RCT	24	26
Key, 2020 [31]	MMAT	Mixed-methods	Yes to all fine	Yes to all
Krishnamohan, 2017 [41]	ICROMS	CBA	18	19
Mabe, 2014 [40]	JBI (Study 1)	Cross-Sectional	Yes to Q1,3,4,7,8 and No to Q2,5,6*	Yes to Q1,3,4,7,8 and No to Q2,5,6
	ICROMS (Study 2)	CBA	22*	23
Pappa, 2017 [32]	MMAT	Mixed-Methods	No to 5.1 and Yes to other 4 criteria	No to 5.1 and Yes to other 4 criteria
Pope, 2019 [29]	ICROMS	RCT	26	24
Raggat, 2018 [42]	JBI	Cross-Sectional	Yes to Q1,2,3,4,7,8 and No to Q5,6*	Yes to Q1,2,3,4,7,8 and No to Q5,6
Torquati, 2018 [33]	MMAT	Mixed-Methods	Yes to all 5	Yes to all 5
Vogel, 2019 [30]	ICROMS	RCT	22	23
Wang, 2020	ICROMS	CBA	22*	21
West, 2016 [37]	ICROMS	CBA	23	23
Wicks, 2020 [38]	JBI (Study 1)	Cross-Sectional	Yes to Q1,3,4,7,8 and No to Q2,5,6*	Yes to Q1,3,4,7,8 and No to Q2,5,6
	ICROMS (Study 2)	CBA	26*	26
Williams, 2019 [35]	ICROMS	NCBA	18	19
Willis, 2016 [36]	ICROMS	NCBA	19	19

Tools Maximum or Minimum Scores – (1) Integrated Quality Criteria for Review of Multiple Study Designs (ICROMS) Minimum Scores: Randomized Controlled Trials (RCT) = 22, Controlled Before-and After (CBA) = 18, Non-Controlled Before-After (NCBA) = 22; (2) Mixed Methods Appraisal Tool (MMAT) for Mixed-Methods: Total of 12 questions answering Yes, No, or Can’t Tell; (3) Joanna Briggs Institute (JBI) Checklist for Analytical Cross Sectional Studies: Total of 8 questions answering Yes, No, or Unclear

### Characteristics of social media interventions

As highlighted in Table 1, there was much heterogeneity across the 18 studies regarding the social media platform used for interventions. Twelve of the 18 studies used Facebook, with 10 of these studies using Private Facebook groups [26–30, 32, 34–36, 40]. The remaining six studies reported on the use of Twitter [33], Reddit [31], WeChat [38], Instagram [37] and the use of multiple social media platforms, where the specific platform used was not specified (although Instagram and Facebook were mentioned) [41].

Information related to how social media was used within the intervention to elicit changes to physical activity and diet varied across the papers. The analysis identified three overarching types of social media use within interventions: (i) interaction – social support, interactions between participants and/or live counselling sessions; (ii) information – the sharing of guidance, advice and educative materials related to physical activity and diet, delivered through text, videos, tailored/personalised content, detailed instructions, an electronic newsletter, notifications and/or reminders; (iii) gamification – encouraging and motivating participants to change physical activity and diet-related behaviours through gamification principles, such as competitions, challenges and rewards. Across all of these types of social media use, none of the studies reported on the use of paid content and/or the use of advertisements and commercial campaigns. However, there is potential that in the studies that explored engagement with established social media communities and groups and/or that focused on general social media use (i.e. time spent on social media), participants could have been exposed to commercial content [31, 37, 39, 41]. All of the 18 studies reported on the uses of interaction, information and gamification in relation to organic content and the content shared between participants and/or by the research team.

Most of the studies (12 of 18) reported on using more than one of the types of social media (i.e. interaction, information, gamification) within the intervention [26, 27, 29–36, 38]. Eight of the 18 studies reported on the use of social media for interaction and information [26, 27, 29, 32, 34–36, 41], of which Facebook was the main platform used. Two studies used a combination of interaction, information and gamification [33, 38], and two used information and gamification [30, 31], and of these studies, three used contemporary mediums of Twitter, WeChat and Reddit and were published between 2017 and 2020. In the Twitter intervention, information involved the sharing of text-based tweets by the intervention team focused on increasing physical activity, increasing fruit and vegetable intake and decreasing sugar sweetened beverage intake, and photo-based tweets of pictures and infographics related to healthy food options and healthy lifestyle tips [33]. Interaction in this particular intervention involved encouraging participants to ask questions within a private Twitter group created specifically for the intervention [33]. In relation to gamification, the participants’ Twitter accounts were linked with a Fitbit tracking device, and individual and group challenges with prizes were developed in relation to physical activity and included step challenges, such as most steps/day or per week, where the results were shared via Twitter [33]. In the intervention that used WeChat for interaction, information and gamification, daily information was shared to participants, discussions were initiated within the community through images of food and exercise and mini exercise challenges were assigned [38]. A similar approach was adopted in the studies that focused on information and gamification [30, 31]. In the intervention that used Reddit, the focus was on the sharing of information related to weight loss, and participants were encouraged to share posts, comment and vote on posts (by liking or disliking posts) within the community [31]. The Facebook intervention involved the sharing of information three times a day and challenges were created in relation to data that could be tracked and shared to Facebook from participants’ Fitbits, and included daily step totals, where prizes for highest number of steps were awarded [30]. Participants were also challenged to share a healthy adaptation of their favourite meal to Facebook [30].

Only a few of the studies (4 of 18) used one type of social media [28, 37, 40]. Two studies focused on information, which involved the researchers posting health education content within Facebook groups two or three times a week [28, 40]. The two further studies focused on interaction through participants sharing images on Instagram [37]. Two studies did not focus on information, interaction or gamification because the focus was on general Facebook whereby participants were encouraged to access Facebook (study 1) and then use Facebook for 20 min (study 2) [39].

### Participant use and engagement with social media

Half of the studies reported on social media use as a measure of intervention engagement. In these studies [26, 31–37, 39], engagement was measured through participation in social media groups, hours spent on social media and/or frequency of views, posts, comments and likes. Between 50 and 100% participation was reported in studies that measured engagement through social media groups and by using metrics related to members joining the group and/or number of views on weekly posts to the group [26, 32, 34]. For studies measuring time spent on social media, engagement ranged from 1 h per day to 2 h per week [37, 39]. In relation to posts, one study reported on the number of tweets made by participants during the intervention and reported marginal differences between an overweight/obese intervention group (233 tweets) vs healthy weight intervention group (208 tweets) [33]. In relation to likes and comments per week, these were relatively similar across the studies reporting on these measures of engagement, and were reported as 3.3 ± 1.4 likes and comments combined per week [36], 1.3 likes per week and 3.2 comments per week [35]. In a cross-sectional study of a natural intervention in an established community on Reddit over a 4-year period, 0.7 ± 1.8 posts per participant and 7.9 ± 34.4 comments per participant were reported [31].

### Methods of data collection

All of the 18 studies measured diet-related outcomes and 10 studies measured both physical activity and diet-related outcomes [26, 28–30, 32–36, 38]. The main method to generate data were through questionnaires (16 of 18) and these included validated surveys for physical activity levels and dietary intake, as well as diary and/or food recall methods [26–30, 32–34, 36–41]. For diet-related outcomes, 10 studies used anthropometric measures (e.g., bioelectrical impedance to estimate body composition; stadiometer to measure height; weighing scale to measure body weight; and tape measure to measure waist circumference) [26–28, 31–35, 38, 40], where 3 of these studies generated data from self-reported weight taken via a weighing scale by the participants [31, 35, 36]. Furthermore, data were generated from blood samples (e.g. glucose test) [27] and an app (e.g. MyFitnessPaL) to document food intake [35]. For physical activity-related outcomes, 8 studies used accelerometers and these included commercial devices (e.g. Fitbits) [26, 28, 30, 32, 33, 35, 36, 39], and 2 studies used fitness tests (e.g. step test) [28, 38].

### Effect of social media interventions on physical activity and diet

As highlighted in Tables 1 and 2, multiple types of study designs were included in this review. The effects of the social media interventions are grouped and described below according to study design.

#### Randomised control trials (RCTs)

Four of the 18 studies were RCTs [26–29], and all four used Facebook and reported on diet-related outcomes. Three of these RCT studies also reported on physical activity outcomes [26–28].

Three studies reported statistically significant increases in physical activity and/or improvements in diet behaviours [26, 27, 29] in relation to moderate-to-vigorous physical activity (MVPA), daily vegetable servings, readiness to increase fruit and vegetable consumption, change in weight (decrease), increased percentage of weight loss, and a reduction in waist circumference, BMI, fat mass and blood cholesterol. In these studies, social media was used for interaction and information.

Three of these studies reported no change or non-statistically significant increases in physical activity and/or diet quality [26–28]. Specifically, there were no changes in steps per day or overall step counts, and non-significant increases in MVPA and self-reported food, fat or alcohol intake. These studies focused on the use of social media for interaction and/or information. One study reported non-statistically significant decreases in physical activity and diet [28] outcomes, related to MVPA, daily energy intake, and vegetable consumption. This study focused on the use of social media for sharing information using private Facebook groups and smartwatches.

#### Non-controlled before and after trials (NCBA)

Three of the 18 studies were NCBA, and all reported on physical activity and diet-related outcomes [33–35]. Two studies used Facebook [34, 35] and one Twitter [33].

All 3 of the NCBA studies reported no change and/or non-statistically significant increases in physical activity and/or diet, for outcomes related to overall step count, fruit and vegetables intake (self-reported), weight loss or weight change, BMI, waist circumference, enjoyment of physical activity and time spent in physical activity. The focus of these interventions varied. Two of these studies focused on the use of social media for interaction and information [34, 35], and the other on interaction, information and gamification [33]. One of the NCBA studies reported non-statistically significant decreases in relation to steps count and sugar-sweetened beverage intake [33]. This study focused on the use of Twitter for interaction, information and gamification over an 8-week intervention.

#### Mixed-methods

Three of the 18 studies were mixed-methods. Two studies reported on physical activity and diet outcomes [30, 32], and one reported on diet outcomes only [31]. Two of the mixed-methods studies used Facebook [30, 32] and one reported on Reddit [31].

Two of the mixed-methods studies reported statistically significant improvements in diet quality. Statistically significant increases in the Healthy Eating Index (HEI) and decreases in Dietary Inflammatory Index scores were reported in a 12-week intervention that used Facebook to share information on diet three times per day [30]. Statistically significant increases in fruit and vegetable intake were reported in a 12-week intervention that used Facebook for information and interaction [32]. However, although this Facebook intervention resulted in improvements to diet quality, statistically significant decreases were reported for physical activity, in relation to MVPA and daily step count.

Three of the mixed methods studies reported no change in diet-related outcomes [30–32], such as no change to BMI, waist circumference or weight loss. These interventions focused on the use of social media for information and gamification [30], interaction, information and gamification [31], and interaction and information [32].

#### Controlled before and after trials (CBA)

Five of the 18 studies were CBA design [36–40]. Two studies reported on physical activity and diet outcomes [38, 40] and three studies reported on diet outcomes only [36, 37, 39]. Three studies used Facebook [36, 39, 40], one study used WeChat [38] and one study used Instagram [37].

Three of the CBA studies reported statistically significant improvements in diet-related behaviours and/or physical activity [36, 38, 40]. For diet, statistically significant improvements were reported in relation to vegetable, fruit, milk and dairy intake [38], weight control strategies (reduced snacking, increased daily self-weighing and graphing of weight and reduced energy and fat intakes) [36], a reduced number of days junk food was consumed per week, and a decrease in BMI [40]. Two studies reported on statistically significant improvements to physical activity indicated by increases in levels of physical activity (changing from low to high) [38], and the steps and miles walked per day [36]. The studies reporting statistically significant improvements to physical activity and diet-related outcomes varied; one study focused on the use of social media for interaction, information and gamification [38], one study focused on information and interaction [36], and the remaining study focused on information only [40]. Statistically significant negative outcomes were associated with social media use in two studies and in relation to disordered eating [37, 39]. In study 2 of Mabe et al. [40], disordered eating was associated with the perceived importance of receiving comments on a status or photos, receiving likes, and comparing photos with female friends [39]. In study 2 of Wicks and Keel [38], significant increases in eating disorder cognitions were reported for individuals who posted and edited their photos on Instagram [37]. Negative outcomes were thus associated with interactions on social media in relation to personal information, such as photos or status.

#### Cross-sectional

Three of the 18 studies were cross-sectional. Two of the studies reported on statistically significant negative outcomes in relation to disordered eating, where a positive correlation was observed in relation to disordered eating between general Facebook use (i.e. time spent on social media) [39] and posting and editing photos on Instagram [37]. One study reported non-statistically significant increases in positive attitudes toward exercise and a healthy diet [41]. This intervention focused on the use of social media for interaction and information, specifically recruiting participants who accessed fitspiration content^1^ across different social media sites (although the authors noted Facebook and Instagram). Results indicated that access to fitspiration content improved attitudes toward exercise and diet in the majority of participants. Reported positive benefits included increased motivation to exercise and eat healthfully, increased engagement with a supportive community, and access to health information that participants perceived to be reliable. A minority of participants experienced negative impacts, including feelings of guilt, inadequacy, and failure to meet goals. It is important to note that psychological distress and risks of an eating disorder or compulsive exercise behaviours were common in the study sample [41].

## Discussion

The studies included in this review broadly indicate that social media interventions can influence positive changes in physical activity and diet-related behaviours, through increases in physical activity levels, healthy modifications to food intake, and beneficial changes to body composition or body weight. Diverse study designs were included in this review – RCTs, NCBAs, CBA, Mixed Methods and Cross-Sectional - where effects ranged between statistically significant and non-significant/no change. Most papers used self-reported questionnaires to measure physical activity and diet-related behaviours, and these have recognised limitations, including unreliable estimates, recall bias and misinterpretation of questions [42, 43]. Overall, only two-thirds of the included studies were assessed to be high quality, with insufficient evidence provided in a third of the studies on the protocol and/or outcome measures. Hence, the evidence from these studies about the effect and direction of social media interventions on physical activity and diet-related behaviours is inconclusive.

The use of Facebook, Facebook groups and the accessibility of information and interaction were the main characteristics of social media interventions used to elicit changes in physical activity and diet-related behaviours. Measures of participant use and engagement with social media also show both passive (viewing posts) and active participation (interactions through sharing posts, likes, and comments) with social media during the interventions. This review has also provided new evidence on the positive influence of contemporary social media sites, including Instagram, Reddit, WeChat and Twitter and their contemporary affordances, such as photo sharing and editing, sub-groups, and group chats. Interventions that used contemporary social media sites tended to focus on multiple types of social media use (i.e. information, interaction and gamification), and often included a focus on gamification, such as through challenges, competitions or rewards. Furthermore, new evidence is provided in this review from studies that reported on the multi-modal uses of social media, whereby data from wearable fitness trackers was connected with participants’ social media accounts [30, 33]. These studies provide important evidence of the positive effects of the contemporary uses of technology [5, 6], in relation to the multi-modal connectivity of smart devices, self-surveillance practices, the automation of actions and the role of gamification in social media interventions [44]. Overall, the new evidence reported in this review suggests that the functionality of social media is diversifying, and this provides increased opportunities to reach and engage diverse groups to positively influence physical activity and diet-related behaviours within social media interventions. The contemporary uses of social media should be explored further and applied to social media interventions to measure and compare effects on physical activity and diet-related behaviours. To measure relationships between social media interventions and health behaviours, we also suggest that future research should improve methods of specifying and reporting the social media intervention, as well as applying established guidance and frameworks for evaluating interventions, such as the UK Medical Research Council’s guidance for complex interventions and/or the Behaviour Change Techniques Taxonomy [45].

The main target population of most studies included in this review was young female adults (aged 18–35) attending college/university, and this finding prevented a comparison of outcomes across different population groups. As such, we were unable to address this proposed aim. None of the included papers reported on adolescents (aged < 16), even though this population group use social media extensively [14], and in relation to physical activity and diet [2]. Some of the papers reported on adults aged 40–60 [27, 30, 32, 35], and this reflects increased rates of social media use in society by this population group [15]. Across the papers there was a lack of consistency in reporting of ethnicity and socio-economic factors, although for the papers where ethnicity was reported, the samples were predominantly white. Evidence on societal uses of social media suggests that rates of use are similar across ethnicities and socio-economic status, although types of social media use may differ [14, 46]. There is therefore a need to design studies that directly assess differences in social media use and the impact of interventions for various ages, genders, ethnic groups, and levels of education and income. Future studies could take the form of targeted interventions for specific groups, as well as recruit large sample sizes that are representative of the population and/or the demographics of societal uses of various types of social media.

## Conclusion

The aims of the review were to address current gaps in evidence on how and why social media interventions influence physical activity and diet-related behaviour change, and to understand the uses and effects of contemporary social media mediums. This review has provided a detailed description of all evidence published since 2014, identifying key characteristics of social media use and the target population of interventions, of which the findings indicate much heterogeneity in study design and outcome measures. Overall, new evidence is provided on the affordances of social media that can be used by policy makers, professionals, organisations and/or researchers to inform the design of future social media interventions to elicit positive changes to physical activity and diet-related behaviours. Further evidence is still required on the use of contemporary social media sites and the contemporary affordances of social media and their effects on physical activity and diet-related behaviours. In addition, this review reveals the need for further methodological rigor to determine the direct effects of social media interventions on physical activity and diet-related behaviours, such as through the use of RCTs, objective measures of physical activity and diet, and the inclusion of representative samples of participants.

## Supplementary Information

**Additional file 1.**

## Data Availability

All data generated or analysed during this study are included in this published article [and its supplementary information files].

## Abbreviations

CBA: Controlled before and after trials
BMI: Body Mass Index
HEI: Healthy Eating Index
ICROMS: Integrated Quality Criteria Review of Multiple Study Designs
MVPA: Moderate-Vigorous Physical Activity
NCBA: Non-Controlled before-after trials
PICOT: Participants, Intervention, Comparison, Outcome, Type of Study
PRISMA: Preferred reporting items for systematic reviews and meta-analyses
WHO: World Health Organisation

## Footnotes

1 Fitness inspiration is often abbreviated to ‘fitspiration’ and this is a popular trend on social media where individuals post or view images, quotes and advice about fitness and nutrition. Some users who follow the fitspiration trend also engage in discussions and shape online fitness cultures about a healthy appearance and optimal dieting and exercise behaviours
