# Supplementary material for: The effect of social media interventions on physical activity and dietary behaviours in young people and adults: a systematic review
Source: Int J Behav Nutr Phys Act. 2021 Jun 5;18:72. doi: 10.1186/s12966-021-01138-3 (PMC8180076; doi:10.1186/s12966-021-01138-3)
Supplement: Supplementary file 1 — Additional file 1. [file 12966_2021_1138_MOESM1_ESM.docx]

**Table 1.** Social Media Systematic Review Search Terms

| **Social Media (1)** | **Physical Activity (2)** | **Diet/Nutrition (3)** | **Age Groups (4)** |
| --- | --- | --- | --- |
| “Social Media” | “Physical Activity*” to capture physical activity, Physical activities | Diet | “Young Adult*” *to capture adult/adults* |
| “Social Network*” *to capture Social network/social networks/social networking* | Exercis* *to capture exercise/ exercising/exercises* | Nutrition | “High School*” to capture high school/s |
| “Instant Messag*” *to capture Instant Messaging, Instant Message/* | Sedentary | “Dietary Behaviour” | Adult* *to capture adult/adults* |
| “Instant Messenger | “Leisure time physical activit*” *to capture activities, activity* | “Weight Management” | “Older Adult*” *to capture adult/adults* |
| Facebook | “Recreational Physical Activit*” *to capture activities, activity* | Food |  |
| Instagram | Workout | “Eating Attitudes” |  |
| WhatsApp | “Subjective physical activit*” *to capture activities, activity* | “Dietary Pattern” |  |
| Twitter | “Objective Physical Activit*” *to capture activities, activity* | Eating |  |
|  |  | Cooking |  |
|  |  | “Eating Behaviour” |  |

**Table 2:** Example of Search String Table for databases (will be used to search title, abstract and keywords)

|  | **Medline(OVID)** | **Embase (OVID)** | **EBSCO** | **Wiley** | **Scopus** |
| --- | --- | --- | --- | --- | --- |
| 1 | (“Social Media”) OR (“Social Network*”) OR “Instant Messag*”) OR (“Instant Messenger”) OR (Facebook) OR (Instagram) OR (WhatsApp) OR (Twitter). kw, ti, ab. | (“Social Media”) OR (“Social Network*”) OR “Instant Messag*”) OR (“Instant Messenger”) OR (Facebook) OR (Instagram) OR (WhatsApp) OR (Twitter). kw, ti, ab. | (“Social Media”) OR (“Social Network*”) OR “Instant Messag*”) OR (“Instant Messenger”) OR (Facebook) OR (Instagram) OR (WhatsApp) OR (Twitter). kw, ti, ab. | (“Social Media” OR “Social Network*” OR “Instant Messag*” OR “Instant Messenger” OR Facebook OR Instagram OR WhatsApp OR Twitter). Kw, ti, ab. | TITLE-ABS-KEY("Social Media" OR "Social Network*" OR "Instant Messag*" OR "Instant Messenger" OR Facebook OR Instagram OR WhatsApp OR Twitter)) |
| 2 | (“Physical Activit*”) OR (Exercis*) OR (Sedentary) OR (“Leisure Time Physical Activit*”) OR (“Recreational Physical Activit*”) OR (Workout) OR (“Subjective Physical Activit*) OR (Objective Physical Activit*). kw, ti, ab. | (“Physical Activit*”) OR (Exercis*) OR (Sedentary) OR (“Leisure Time Physical Activit*”) OR (“Recreational Physical Activit*”) OR (Workout) OR (“Subjective Physical Activit*) OR (Objective Physical Activit*). kw, ti, ab. | (“Physical Activit*”) OR (Exercis*) OR (Sedentary) OR (“Leisure Time Physical Activit*”) OR (“Recreational Physical Activit*”) OR (Workout) OR (“Subjective Physical Activit*) OR (Objective Physical Activit*). kw, ti, ab. | (“Physical Activit*” OR Exercis* OR Sedentary OR “Leisure Time Physical Activit*” OR “Recreational Physical Activit*” OR Workout OR “Subjective Physical Activit* OR Objective Physical Activit*). Kw, ti, ab. | TITLE-ABS-KEY(“Physical Activit*” OR Exercis* OR Sedentary OR “Leisure Time Physical Activit*” OR “Recreational Physical Activit*” OR Workout OR “Subjective Physical Activit* OR Objective Physical Activit*). kw, ti, ab. |
| 3 | 1 and 2 = 874 | 1 and 2 = 1942 | 1 and 2 = 391 | 1 and 2 = 32 | 1 and 2 = 3742 |
| 4 | (Diet) OR (Nutrition) OR (“Dietary Behaviour”) OR (“Dietary Behavior”) OR (“Weight Management”) OR (Food) OR (“Eating Attitudes”) OR (“Dietary Pattern”) OR (Eating) OR (Cooking) OR (“Eating Behaviour”) OR (“Eating Behavior”) kw, ti, ab. | (Diet) OR (Nutrition) OR (“Dietary Behaviour”) OR (“Dietary Behavior”) OR (“Weight Management”) OR (Food) OR (“Eating Attitudes”) OR (“Dietary Pattern”) OR (Eating) OR (Cooking) OR (“Eating Behaviour”) OR (“Eating Behavior”) kw, ti, ab. | (Diet) OR (Nutrition) OR (“Dietary Behaviour”) OR (“Dietary Behavior”) OR (“Weight Management”) OR (Food) OR (“Eating Attitudes”) OR (“Dietary Pattern”) OR (Eating) OR (Cooking) OR (“Eating Behaviour”) OR (“Eating Behavior”) kw, ti, ab. | (Diet OR Nutrition OR “Dietary Behaviour” OR “Dietary Behavior” OR “Weight Management” OR Food OR “Eating Attitudes” OR “Dietary Pattern” OR Eating OR Cooking OR “Eating Behaviour” OR “Eating Behavior”). Kw, ti, ab. | TITLE-ABS-KEY(Diet OR “Dietary Behaviour” OR "Dietary Behavior” OR "Weight Management" OR Food OR “Eating Attitudes” OR “Dietary Pattern” OR Eating OR Cooking OR “Eating Behaviour” OR “Eating Behavior”) |
| 5 | 1 and 4 = 1109 | 1 and 4 = 2290 | 1 and 4 = 470 | 1 and 4 = 12 | 1 and 4 = 5021 |
| 6 | 1 and 2 and 4 = 265 | 1 and 2 and 4 = 619 | 1 and 2 and 4 = 47 | 1 and 2 and 4 = 13 | 1 and 2 and 4 = 773 |
| 7 | (“Young Adult*”) OR (“High School*”) OR (Adult*) OR (“Older Adult*”). kw, ti, ab. | (“Young Adult*”) OR (“High School*”) OR (Adult*) OR (“Older Adult*”). kw, ti, ab. | (“Young Adult*”) OR (“High School*”) OR (Adult*) OR (“Older Adult*”).ti, ab. | (“Young Adult*” OR “High School*” OR Adult* OR “Older Adult*”). Kw, ti, ab. | TITLE-ABS-KEY("Young Adult" OR "High School*" OR Adult* OR "Older Adult*") |
| 8 | 1 and 2 and 4 and 7 = 78 | 1 and 2 and 4 and 7 = 157 | 1 and 2 and 4 and 7 = 21 | 1 and 2 and 4 and 7 = 15 | 1 and 2 and 4 and 7 = 355 |
| 9 | Limiters – Publication date 2014 onwards and English language | Limiters – Publication date 2014 onwards and English language | Limiters – Publication date 2014 onwards and English language | Limiters – Publication date 2014 onwards and English language | Limiters – Publication date 2014 onwards and English language |
| 10 | 1 and 2 and 9 = 581 | 1 and 2 and 9 = 1453 | 1 and 2 and 9 = 246 | 1 and 2 and 9 = 25 | 1 and 2 and 9 = 2751 |
| 11 | 1 and 4 and 9 = 779 | 1 and 4 and 9= 1759 | 1 and 4 and 9 = 366 | 1 and 4 and 9 = 9 | 1 and 4 and 9 = 3793 |
| 12 | 1 and 2 and 4 and 9 = 187 | 1 and 2 and 4 and 9 = 466 | 1 and 2 and 4 and 9 = 29 | 1 and 2 and 4 and 9 = 12 | 1 and 2 and 4 and 9 = 867 |
| 13 | 1 and 2 and 4 and 7 and 9 = 59 | 1 and 2 and 4 and 7 and 9 = 116 | 1 and 2 and 4 and 7 and 9 = | 1 and 2 and 4 and 7 and 9 = 9 | 1 and 2 and 4 and 7 and 9 = 396 |
|  | **n without age groups = 1872** | **n without age groups = 466** | **n = without age groups = 29** | **n without age groups = 12** | **n without age groups = 625** |
|  | **n with age groups = 59** | **n with age groups = 116** | **N with age groups = 12** | **n with age groups = 9** | **n with age groups = 303** |
| **Total of studies (Social Media + Physical Activity + Diet + Age) once combined and duplicates/grey literature removed = 356** | | | | | |

**Table 3:** Re-run (Feb 2021)) of Search String Table for databases (will be used to search title, abstract and keywords)

|  | **Medline(OVID)** | **Embase (OVID)** | **EBSCO** | **Wiley** | **Scopus** |
| --- | --- | --- | --- | --- | --- |
| 1 | (“Social Media”) OR (“Social Network*”) OR “Instant Messag*”) OR (“Instant Messenger”) OR (Facebook) OR (Instagram) OR (WhatsApp) OR (Twitter). kw, ti, ab. | (“Social Media”) OR (“Social Network*”) OR “Instant Messag*”) OR (“Instant Messenger”) OR (Facebook) OR (Instagram) OR (WhatsApp) OR (Twitter). kw, ti, ab. | (“Social Media”) OR (“Social Network*”) OR “Instant Messag*”) OR (“Instant Messenger”) OR (Facebook) OR (Instagram) OR (WhatsApp) OR (Twitter). kw, ti, ab. | (“Social Media” OR “Social Network*” OR “Instant Messag*” OR “Instant Messenger” OR Facebook OR Instagram OR WhatsApp OR Twitter). Kw, ti, ab. | TITLE-ABS-KEY("Social Media" OR "Social Network*" OR "Instant Messag*" OR "Instant Messenger" OR Facebook OR Instagram OR WhatsApp OR Twitter)) |
| 2 | (“Physical Activit*”) OR (Exercis*) OR (Sedentary) OR (“Leisure Time Physical Activit*”) OR (“Recreational Physical Activit*”) OR (Workout) OR (“Subjective Physical Activit*) OR (Objective Physical Activit*). kw, ti, ab. | (“Physical Activit*”) OR (Exercis*) OR (Sedentary) OR (“Leisure Time Physical Activit*”) OR (“Recreational Physical Activit*”) OR (Workout) OR (“Subjective Physical Activit*) OR (Objective Physical Activit*). kw, ti, ab. | (“Physical Activit*”) OR (Exercis*) OR (Sedentary) OR (“Leisure Time Physical Activit*”) OR (“Recreational Physical Activit*”) OR (Workout) OR (“Subjective Physical Activit*) OR (Objective Physical Activit*). kw, ti, ab. | (“Physical Activit*” OR Exercis* OR Sedentary OR “Leisure Time Physical Activit*” OR “Recreational Physical Activit*” OR Workout OR “Subjective Physical Activit* OR Objective Physical Activit*). Kw, ti, ab. | TITLE-ABS-KEY(“Physical Activit*” OR Exercis* OR Sedentary OR “Leisure Time Physical Activit*” OR “Recreational Physical Activit*” OR Workout OR “Subjective Physical Activit* OR Objective Physical Activit*). kw, ti, ab. |
| 4 | (Diet) OR (Nutrition) OR (“Dietary Behaviour”) OR (“Dietary Behavior”) OR (“Weight Management”) OR (Food) OR (“Eating Attitudes”) OR (“Dietary Pattern”) OR (Eating) OR (Cooking) OR (“Eating Behaviour”) OR (“Eating Behavior”) kw, ti, ab. | (Diet) OR (Nutrition) OR (“Dietary Behaviour”) OR (“Dietary Behavior”) OR (“Weight Management”) OR (Food) OR (“Eating Attitudes”) OR (“Dietary Pattern”) OR (Eating) OR (Cooking) OR (“Eating Behaviour”) OR (“Eating Behavior”) kw, ti, ab. | (Diet) OR (Nutrition) OR (“Dietary Behaviour”) OR (“Dietary Behavior”) OR (“Weight Management”) OR (Food) OR (“Eating Attitudes”) OR (“Dietary Pattern”) OR (Eating) OR (Cooking) OR (“Eating Behaviour”) OR (“Eating Behavior”) kw, ti, ab. | (Diet OR Nutrition OR “Dietary Behaviour” OR “Dietary Behavior” OR “Weight Management” OR Food OR “Eating Attitudes” OR “Dietary Pattern” OR Eating OR Cooking OR “Eating Behaviour” OR “Eating Behavior”). Kw, ti, ab. | TITLE-ABS-KEY(Diet OR “Dietary Behaviour” OR "Dietary Behavior” OR "Weight Management" OR Food OR “Eating Attitudes” OR “Dietary Pattern” OR Eating OR Cooking OR “Eating Behaviour” OR “Eating Behavior”) |
| 7 | (“Young Adult*”) OR (“High School*”) OR (Adult*) OR (“Older Adult*”). kw, ti, ab. | (“Young Adult*”) OR (“High School*”) OR (Adult*) OR (“Older Adult*”). kw, ti, ab. | (“Young Adult*”) OR (“High School*”) OR (Adult*) OR (“Older Adult*”).ti, ab. | (“Young Adult*” OR “High School*” OR Adult* OR “Older Adult*”). Kw, ti, ab. | TITLE-ABS-KEY("Young Adult" OR "High School*" OR Adult* OR "Older Adult*") |
| 9 | Limiters – Publication date 2020-Current onwards and English language | Limiters – Publication date 2020-Current onwards and English language | Limiters – Publication date 2020-Current onwards and English language | Limiters – Publication date 2020-Current onwards and English language | Limiters – Publication date 2020-Current onwards and English language |
|  | **n with age groups = 13** | **n with age groups = 133** | **N with age groups = 2** | **n with age groups = 0** | **n with age groups = 18** |
| **Total of studies (Social Media + Physical Activity + Diet + Age) once combined and duplicates/grey literature removed = 166** | | | | | |

**Table 4.** Quality Assessment Outcomes for Included Studies

| **Author, Year** | **Tool Used** | **Quality Rating** | **Quality Appraisal Outcomes** | **Include/Exclude** |
| --- | --- | --- | --- | --- |
| Ashton, 2017 | ICROMS (RCT) | Scored higher than minimum global score of 22 | Study was ‘unclear’ or did not provide sufficient information on:   - The follow-up of subjects, patients or episodes of care - If the data collection was unaffected by the intervention or how this was mitigated - If ethical issues were adequately addressed | Include |
| Jane, 2017 |  |  | Study was ‘unclear’ or did not provide sufficient information on:   - Managing bias in blinding - If outcome measures were assessed blindly to protect against bias - The follow-up of subjects, patients or episodes of care - If the data collection was unaffected by the intervention or how this was mitigated. |  |
| Pope, 2019 |  |  | Study was ‘unclear’ or did not provide sufficient information on:   - Managing bias in blinding - Whether primary outcome measures were assessed blindly - Follow-up of subjects, patients and episodes of care - If the data collection was unaffected by the intervention or how this was mitigated. |  |
| Vogel, 2019 |  |  | Study was ‘unclear’ or did not provide sufficient information on:   - Managing bias in blinding - Whether primary outcome measures were assessed blindly - If the sequence generation for managing bias in sampling was done adequately - If the allocation was adequately concealed - Follow-up of subjects, patients or episodes of care - If the data collection was unaffected by the intervention or how this was mitigated - If the study is free of selective outcome reporting. |  |
| Chung, 2017 | ICROMS (NCBA) | Scored lower than minimum global score of 22 | Study was ‘unclear’ or did not provide sufficient information on:   - A clear statement of the aims or goals of the research - Reporting of adequate baseline measurements - Justification or clear rationale for sample choice - Whether blinded assessment of primary outcome measures were completed to protect against detection bias - If reliable primary outcome measures were used - If the data collection was unaffected by the intervention or how this was mitigated - If ethical issues were adequately addressed. | Exclude |
| Williams, 2019 |  |  | Study was ‘unclear’ or did not provide sufficient information on:   - Reporting of adequate baseline measurements - Justification or clear rationale for sample choice - Whether blinded assessment of primary outcome measures were completed to protect against detection bias - If reliable primary outcome measures were used - If the data collection was unaffected by the intervention or how this was mitigated - If ethical issues were adequately addressed   The study did not provide an adequate explanation or justification for lack of a control group. |  |
| Willis, 2016 |  |  | Study was ‘unclear’ or did not provide sufficient information on:   - Adequate baseline measurements - Justification or clear rationale for sample choice - Whether blinded assessment of primary outcome measures were completed to protect against detection bias - If reliable primary outcome measures were used - If the data collection was unaffected by the intervention or how this was mitigated - If ethical issues were adeqautely addressed.   The study did not provide an adequate explanation or justification for lack of a control group. |  |
| Krishnamohan, 2017 | ICROMS (CBA) | Scored higher than minimum global score of 18 | Study was ‘unclear’ or did not provide sufficient information on:   - A clear statement of the aims or goals of the research - A clear rational for selection or allocation of participants to intervention or control groups - Baseline measures inadequately reported - Unclear protection against contamination between groups - Whether blinded assessment of primary outcome measures were completed to protect against detection bias - If incomplete outcome data adequately addressed - A rational explanation for the shape of intervention effect - Limitations were not reasonably considered in interpretation of outcomes - If ethical issues were adequately addressed | Include |
| Wang, 2020 |  |  | Study was ‘unclear’ or did not provide sufficient information on:   - Baseline measures inadequately reported - Investigators did not ensure the control group did not receive the intervention (communication was likely) - Outcomes were not assessed blindly or variables not objective - Incomplete outcome data was no addressed adequately - The effect of intervention on data collection was unclear |  |
| West, 2016 |  |  | Study was ‘unclear’ or did not provide sufficient information on:   - A clear statement of the aims or goals of the research - A clear rational for selection or allocation of participants to intervention or control groups - Unclear protection against contamination between groups - Whether blinded assessment of primary outcome measures were completed to protect against detection bias - If incomplete outcome data adequately addressed - A rational explanation for the shape of intervention effect - Limitations were not reasonably considered in interpretation of outcomes - If ethical issues were adequately addressed |  |
|  | | | | |
| Mabe, 2014 | JBI (Cross-Sectional)- Study 1 | Answered ‘No’ to 3 out of 8 Questions | Study did not describe in detail the subjects or setting, identify confounding factors or provide strategies to deal with confounding factors | Include |
| Wicks, 2020 |  |  |  |  |
| Mabe, 2014 | ICROMS (CBA) – Study 2 | Scored higher than minimum global score of 18 | Study was ‘unclear’ or did not provide sufficient information on:   - Investigators did not ensure the control group did not receive the intervention (communication was likely) - Outcomes were not assessed blindly or variables not objective - Incomplete outcome data not addressed adequately - The effect of intervention on data collection was unclear | Include |
| Wicks, 2020 |  |  | Study was ‘unclear’ or did not provide sufficient information on:   - Investigators did not ensure the control group did not receive the intervention (communication was likely) - Outcomes were not assessed blindly or variables not objective |  |
|  | | | | |
| Key, 2020 | MMAT (Mixed-methods) | ‘Yes’ to all criteria | Study answered ‘Yes’ to all criteria for mixed-methods | Include |
| Torquati, 2018 |  |  |  |  |
| Pappa, 2017 |  | ‘No’ to one criteria and ‘Yes’ to all other criteria. | Study did not provide adequate rationale for using a mixed methods design to address the research question. |  |
|  | | | | |
| Raggat, 2018 | JBI (Cross-sectional) | Answers ‘no’ to 2 out of 8 questions. | Study did not identify confounding factors or provide strategies to deal with confounding factors | Include |

***Tools Maximum or Minimum Scores*** *– (1) Integrated Quality Criteria for Review of Multiple Study Designs(ICROMS) Minimum Scores: Randomized Controlled Trials (RCT) = 22, Controlled Before-and After (CBA) = 18, Non-Controlled Before-After (NCBA) = 22; (2) Mixed Methods Appraisal Tool (MMAT) for Mixed-Methods: Total of 12 questions answering Yes, No, or Can’t Tell; (3) Joanna Briggs Institute (JBI) Checklist for Analytical Cross Sectional Studies: Total of 8 questions answering Yes, No, or Unclear*.
